# Sec61β facilitates the maintenance of endoplasmic reticulum homeostasis by associating microtubules

**DOI:** 10.1007/s13238-017-0492-5

**Published:** 2017-11-22

**Authors:** Yimeng Zhu, Gangming Zhang, Shaoyu Lin, Juanming Shi, Hong Zhang, Junjie Hu

**Affiliations:** 1 National Laboratory of Biomacromolecules, CAS Center for Excellence in Biomacromolecules, Institute of Biophysics, Chinese Academy of Sciences, Beijing, 100101 China; 2 Department of Genetics and Cell Biology, College of Life Sciences, Nankai University, Tianjin, 300071 China; 3 Present Address: Programs in Biomedical and Biological Sciences, University of Southern California, Los Angeles, CA 90089 USA

**Keywords:** ER stress, Microtubule, Sec61β, Translocon, Ribosome

## Abstract

Sec61β, a subunit of the Sec61 translocon complex, is not essential in yeast and commonly used as a marker of endoplasmic reticulum (ER). In higher eukaryotes, such as *Drosophila*, deletion of Sec61β causes lethality, but its physiological role is unclear. Here, we show that Sec61β interacts directly with microtubules. Overexpression of Sec61β containing small epitope tags, but not a RFP tag, induces dramatic bundling of the ER and microtubule. A basic region in the cytosolic domain of Sec61β is critical for microtubule association. Depletion of Sec61β induces ER stress in both mammalian cells and *Caenorhabditis elegans*, and subsequent restoration of ER homeostasis correlates with the microtubule binding ability of Sec61β. Loss of Sec61β causes increased mobility of translocon complexes and reduced level of membrane-bound ribosomes. These results suggest that Sec61β may stabilize protein translocation by linking translocon complex to microtubule and provide insight into the physiological function of ER-microtubule interaction.

**Electronic supplementary material:**

The online version of this article (10.1007/s13238-017-0492-5) contains supplementary material, which is available to authorized users.

## Introduction

In eukaryotic cells, the endoplasmic reticulum (ER) is composed of membrane tubules and sheets (Shibata et al., 2006). Tubules are 30–50 nm in diameter and have a high membrane curvature at their cross-section (Hu et al., 2011), which is stabilized by a class of integral membrane proteins including reticulons and DP1/Yop1p (Hu et al., 2008; Voeltz et al., 2006). In contrast, sheets are formed by parallel membranes ~50 nm apart (Barlowe, 2010). The cisternal spacing is regulated by Climp63 (Shibata et al., 2010), which is proposed to be a luminal bridge, and the surface of the sheets are kept flat, likely by kinectin and p180, which scaffold the membrane as a rod-like structure (Shibata et al., 2010; Zhang and Hu, 2016).

The distinct ER shapes are thought to carry out different functions. Tubules are likely involved in membrane trafficking, lipid metabolism, organelle contact, and stress sensing (Wang et al., 2017), whereas sheets are mostly locations for protein synthesis (Shibata et al., 2006; Voeltz et al., 2002). Translating polysomes for ER-targeting proteins prefer the more accommodating surface of ER sheets, and their abundance could dictate the amount of ER sheets (Shibata et al., 2010). Nascent polypeptides, when exiting ribosomes, need to traverse ER membranes through a channel known as the Sec61 complex or translocon (Rapoport, 2007). Therefore, Sec61 and its associating proteins are enriched in ER sheets (Shibata et al., 2010).

Newly synthesized proteins are modified and folded in the ER. If misfolded proteins accumulate in the ER, they induce ER stress and activate signaling events known as the unfolded protein response (UPR) (Bernales et al., 2006; Ron and Walter, 2007; Schroder and Kaufman, 2005; Walter and Ron, 2011). The UPR consists of three signaling arms: IRE1, PERK, and ATF6. Initially, protective efforts are made, such as increasing chaperones and decreasing translations. Activated IRE1 splices *XBP1* mRNA, which is translated into an active transcription factor that up-regulates the levels of chaperones (Calfon et al., 2002; Lee et al., 2002; Yoshida et al., 2001). PERK phosphorylates eIF2α and inhibits translation so that the burden on the ER can be relieved (Harding et al., 2000; Harding et al., 1999), and ATF6 is cleaved into a soluble transcription factor that also helps deal with ER stress (Haze et al., 1999; Yoshida et al., 2001). If the imbalance of proteostasis in the ER is sustained, programmed cell death is triggered (Tabas and Ron, 2011). Indicators for UPR are often used as markers of ER health.

Like many other organelles, the ER, in the form of either tubules or sheets, is closely associated with microtubules (Friedman and Voeltz, 2011; Staehelin, 1997; Terasaki et al., 1986; Voeltz et al., 2002). The microtubule cytoskeleton not only helps position and support ER membranes, but also actively participates in remodeling the ER (Wang et al., 2013). ER tubules are constantly pulled out of existing ER membranes, by either associating with the growing plus end of the microtubule or sliding along the microtubule with molecular motors (Friedman and Voeltz, 2011). When microtubules are depolymerized by nocodazole treatment, the peripheral tubular ER network retracts towards the center of the cell, yielding an ER made up of mostly sheets (Terasaki et al., 1986). Thus, the microtubule network plays an important role in ER morphogenesis.

Several ER-resident proteins that associate with microtubules have been identified. STIM1 binds to the microtubule plus end, moving it closer to its interaction partner on the plasma membrane, Orai (Carrasco and Meyer, 2011; Grigoriev et al., 2008; Park et al., 2009). Tubule-localized REEP1, a homolog of DP1 (also known as REEP5), has a C-terminal microtubule-binding domain, the loss of which causes hereditary spastic paraplegia (Park et al., 2010). ER sheet marker Climp63 also engages microtubules using its cytosolic N-terminus in a phosphorylation-dependent manner (Klopfenstein et al., 1998; Vedrenne et al., 2005). Disruption of the interaction likely alters the mobility of sheet-localized translocon complex (Nikonov et al., 2007). These findings suggest that the ER-microtubule association has physiological importance. However, the specific roles of such association are not clear.

As described here, we accidently discovered that Sec61β, the β subunit of the Sec61 translocon complex, interacts directly with microtubules. In the translocon, the α subunit is the pore-forming component, the γ subunit hinges the α subunit, and the transmembrane (TM) domain of the β subunit attaches in the periphery of the channel. The bacterial homolog of Sec61β is non-essential (Rapoport, 2007). In yeast, double deletion of the two Sec61β homologs (Sbh1p and Sbh2p) only causes a temperature-sensitive growth defect and can be rescued by the TM domain of the protein (Feng et al., 2007; Finke et al., 1996). Therefore, Sec61β is better known as a frequently used ER marker (Shibata et al., 2006; Voeltz et al., 2006; Voeltz et al., 2002; Zurek et al., 2011) and a model substrate for tail-anchored insertion into the ER (Abell et al., 2004; Favaloro et al., 2008; Stefanovic and Hegde, 2007). Interestingly, Sec61β is essential for *Drosophila* development (Valcarcel et al., 1999) and critical for *C*. *elegans* development. Depletion of Sec61β in mammalian cells and *C*. *elegans* induces mild ER stress. The microtubule-binding ability of Sec61β is associated with the maintenance of ER homeostasis.

## Results

### Overexpressed Sec61β bundles ER and microtubule

**Figure 1:**
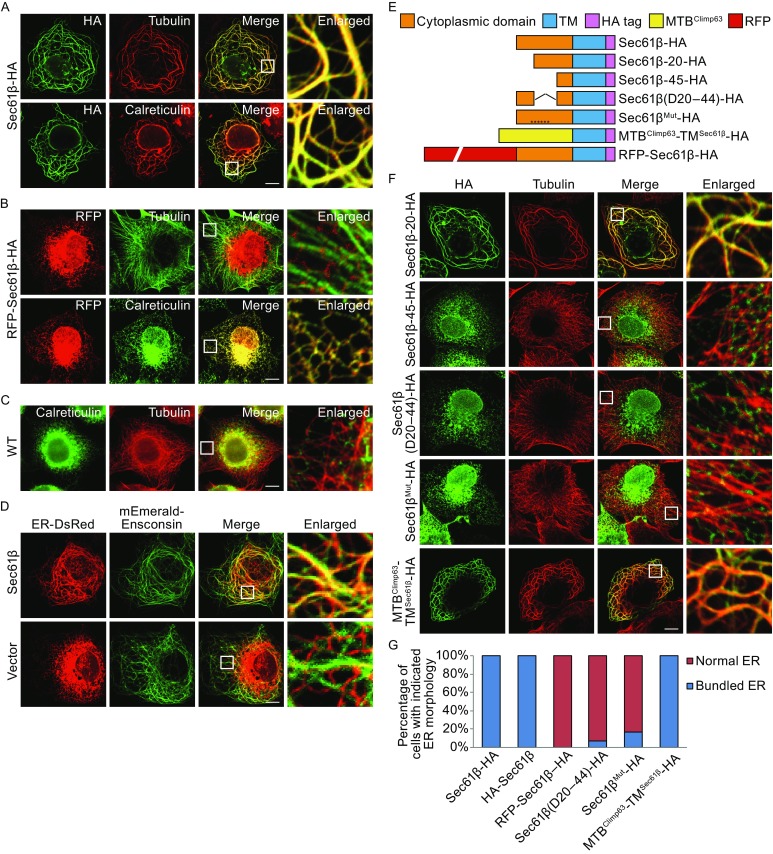
**Overexpressed Sec61β induces bundling of the ER and microtubule**. (A) COS-7 cells transfected with Sec61β-HA were immunostained for HA (green) and endogenous tubulin or luminal ER protein, calreticulin (red), and visualized by fluorescent confocal microscopy. Insets show the enlargement of the indicated area. (B) As in (A), but with RFP-Sec61β-HA and were immunostained only for tubulin or calreticulin (green). (C) As in (A), but wild type COS-7 cells were immunostained for calreticulin (green) and tubulin (red). (D) COS-7 cells co-transfected with Sec61β, ER-DsRed (red), and mEmerald-Ensconsin (green) were visualized live by fluorescent confocal microscopy. (E) Domain structures of constructs. (F) As in (A), but with various constructs shown in (E), and were immunostained for HA-epitope (green) and tubulin (red). (G) The ER morphology of samples shown in (A) and (B) was categorized as “bundled” and “normal” respectively. A total of 150 cells were counted for each sample. All graphs were representative of three repetitions. In (A–D) and (F), scale bars are 10 μm

In the process of making various ER markers, we noticed that Sec61β containing a C-terminal HA tag localizes properly in the ER, but its expression dramatically alters the ER morphology (Fig. 1A and 1G); most of the ER becomes swirl-like thick tubules. Similar ER patterns have been seen in cells overexpressing ER-bound proteins that can interact with microtubules (Klopfenstein et al., 1998; Miyazaki et al., 2012; Park et al., 2010). When tubulin was visualized in COS-7 cells expressing Sec61β-HA, the microtubule network was drastically rearranged and completely overlapped with the ER network, suggesting an augmented association. The same phenomenon was seen when the HA tag was placed on the N-terminus of Sec61β (Figs. S1A and 1G). Conversely, in cells expressing N-terminal RFP-tagged Sec61β, a frequently used version of the ER marker, no ER-microtubule bundling was observed (Fig. 1B and 1G), similar to untreated cells (Fig. 1C). To rule out artifacts that might be introduced by protein tagging or cell fixation, we co-transfected non-tagged Sec61β, ER-DsRed and mEmerald-Ensconsin (microtubule binding protein) into COS-7 cells. Live cell imaging showed same ER-microtubule bundling in transfected cells (Fig. 1D). These results suggest that Sec61β may interact with microtubules and the binding can be prevented with a large tag at the N-terminus.

As a tail-anchored protein, human Sec61β contains an N-terminal cytosolic domain (cytSec61β) of 70 amino acids (Fig. S1B). To pinpoint the microtubule-associating region in Sec61β, we performed a serial truncation of the domain (Fig. 1E). When the first 19 residues were deleted, the Sec61β mutant was still able to bundle microtubules with the ER (Fig. 1F). However, when the deletion was extended to 44 residues, such defects were no longer seen in the ER and microtubule morphology (Fig. 1F). We then removed residues 20–44 of cytSec61β and found that the mutant failed to tangle the ER and microtubules (Fig. 1F and 1G). The region of residues 20–44 is enriched with positively charged amino acids. When all six of them (K20, R25, R32, R34, K35, and R42) were substituted by alanine, the mutant Sec61β-HA no longer linked microtubule to the ER (Fig. 1F and 1G). In contrast and as expected, when we replaced cytSec61β with a known microtubule-binding (MTB) fragment (residues 1–80 of Climp63), the chimera MTB^Climp63^-TM^Sec61β^ behaved the same as Sec61β-HA in gluing the ER and microtubules into swirls (Fig. 1F and 1G). Like all of the truncation mutants, the chimera localized specifically to the ER (Fig. S1C). Notably, alterations of the cytSec61β caused decreased expression level (Fig. S1D). These results suggest that the microtubule-binding site of Sec61β is within a middle region of its cytosolic domain.

### Sec61β interacts directly with tubulin

**Figure 2:**
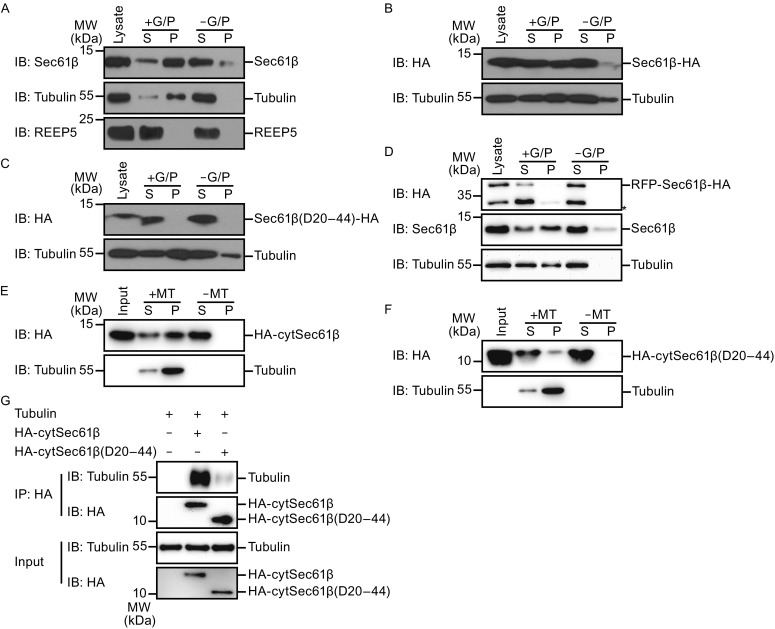
**Sec61β interacts with microtubule**. (A) COS-7 cells were harvested for microtubule co-sedimentation in the absence or presence of GTP/paclitaxel (G/P). Samples of the supernatant (S) and pellet (P) were analyzed by Western blotting. (B–D) As in (A), but transfected with full length Sec61β (B), Sec61β lacking residues 20–44 (C) or RFP-tagged Sec61β (D). The bands with asterisk (*) may be degraded RFP-Sec61β-HA. (E) Purified wild-type HA-cytSec61β was incubated with microtubules (MT) or not, for microtubule co-sedimentation. Samples were analyzed by Western blotting. (F) As in (E), but with HA-cytSec61β lacking residues 20–44. (G) Purified wild-type HA cytSec61β or HA-cytSec61β lacking residues 20–44 was incubated with tubulins and precipitated with anti-HA antibody. The levels of indicated proteins were analyzed with Western blotting

To confirm the interactions between Sec61β and microtubules, we performed microtubule sedimentation assays. Endogenous Sec61β co-precipitated with tubulins when the microtubules were reassembled in COS-7 cell lysates in the presence of GTP and paclitaxel, and precipitated in a glycerol cushion (Fig. 2A). Over-expressed wild-type Sec61β-HA (Fig. 2B), but not the Δ20–44 mutant (Fig. 2C), also co-sedimented with polymerized microtubule. Consistently, RFP-Sec61β failed to settle with microtubules, even when endogenous Sec61β did in the same lysates (Fig. 2D). These results confirm that Sec61β interacts with microtubules.

To test whether the association is direct, we used purified proteins to perform microtubule sedimentation assays. Wild-type HA-cytSec61β was efficiently precipitated by microtubules assembled with purified tubulins (Fig. 2E). The deletion of residues 20–44 again disrupted the interactions (Fig. 2F). Furthermore, when wild-type HA-cytSec61β but not the Δ20–44 mutant, was incubated with tubulins, anti-HA antibodies precipitated tubulins (Fig. 2G). These results suggest that Sec61β engages tubulin directly in an assembly-independent manner.

### Depletion of Sec61β causes ER stress

**Figure 3:**
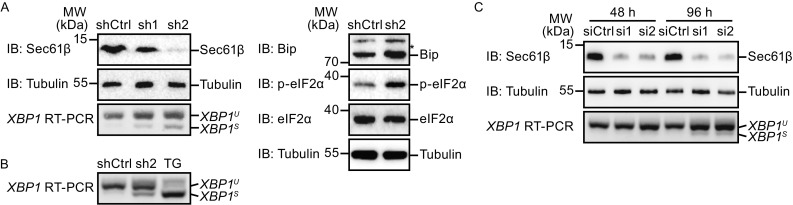
**Depletion of Sec61β triggers ER stress**. (A) COS-7 cells were infected with shRNA-expressing viruses. The levels of Sec61β were determined by Western blotting, and the unspliced (U) *XBP1* and spliced (S) *XBP1* by RT-PCR of *XBP1* mRNA were resolved by agarose gel. ER stress markers, including Bip and phosphorylated eIF2α, were analyzed by Western blotting. Asterisk (*) indicates a nonspecific band. (B) The unspliced (U) *XBP1* and spliced (S) *XBP1* by RT-PCR of *XBP1* mRNA from infected cells or thapsigargin (TG) treated cells were resolved by agarose gel. (C) U2OS cells were transfected with siRNAs of Sec61β for 48 h or 96 h. The levels of Sec61β were determined by Western blotting, and the unspliced *XBP1* and spliced *XBP1* by RT-PCR of *XBP1* mRNA were resolved by agarose gel

To assess the role of Sec61β-mediated microtubule association, we depleted Sec61β using RNA interference. Two different shRNAs against Sec61β were individually introduced into COS-7 cells using a viral vector. Sec61β depletion was detected in both samples, with shRNA #2 being more efficient than #1 (Fig. 3A). However, no obvious morphological ER defects were seen in these cells (Fig. S2A). The same applied to microtubules, Golgi, and mitochondria (Fig. S2A). These results coincide with the redundancy of ER-microtubule interactions.

Next, because Sec61β, as a component of the ER translocon, is associated with the protein synthesis pathway, we tested whether its depletion affects protein homeostasis in the ER. Defective protein production in the ER activates UPR signaling. In Sec61β-depleted COS-7 cells, splicing of *XBP1* mRNA (indicative of IRE1 activation) was detected (Fig. 3A). The level of splicing was more prominent when Sec61β was more efficiently depleted, but overall was moderate compared to that triggered by thapsigargin (TG) treatment (Fig. 3B). Similarly, eIF2α phosphorylation (indicative of PERK activation) was elevated when Sec61β was knocked down (Fig. 3A). *XBP1* splicing was also observed when Sec61β was depleted with siRNAs instead of shRNAs (Fig. 3C). In contrast, depletion of ER tubule marker REEP1 or sheet marker Climp63 did not cause detectable ER stress (Fig. S2C–E), even though both of them interact with microtubules. These data demonstrate that the loss of Sec61β induces ER stress.

We also tested whether Sec61β regulates ER homeostasis in the context of a multicellular organism. Y38F2AR.9 is the *C*. *elegans* homolog of Sec61β. A CHERRY::Y38F2AR.9 reporter showed that, at the larval stage, Y38F2AR.9 formed a reticular network and accumulated around the nucleus, resembling the pattern of the ER (Fig. 5A). In addition, CHERRY::Y38F2AR.9 co-localized with ER marker GFP::TRAM-1, confirming that Y38F2AR.9 has the same localization as Sec61β (Fig. 5A). As in *Drosophila* (Valcarcel et al., 1999), deletion of Y38F2AR.9 caused lethality. Thus, we used RNAi to deplete Y38F2AR.9. Consistently, loss of Y38F2AR.9 caused a significant increase in P*hsp-4*::*GFP* expression (Fig. 5B), indicative of the induction of ER stress. These results confirm that the function of Sec61β is essential and conserved in *C*. *elegans*.

### Microtubule association by Sec61β regulates ER homeostasis

**Figure 4:**
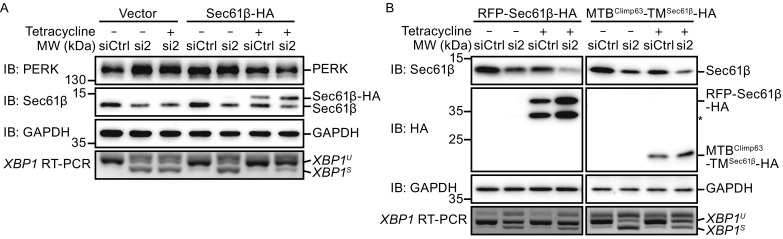
**Sec61β-regulated ER homeostasis requires microtubule binding**. (A) SiRNA transfected Flp-In-293 cells were treated with tetracycline as indicated. The samples were analyzed by Western blotting and agarose gel. (B) As in (A), but in cells expressing MTB^Climp63^-TM^Sec61β^-HA and RFP-Sec61β-HA respectively. The bands with asterisk (*) may be degraded RFP-Sec61β-HA

To probe the role of Sec61β in the maintenance of ER homeostasis, we tested whether Sec61β-mediated microtubule association is critical. To this end, we generated Flp-In-293 cells to stably express wild-type or mutant Sec61β (Fig. S2F). Taken expression levels into consideration (Fig. S1D), we chose two representative mutants: MTB^Climp63^-TM^Sec61β^ (microtubule-binding positive) and RFP-Sec61β (microtubule-binding negative). The ectopic expression of Sec61β was siRNA-resistant and induced by the addition of tetracycline. Meanwhile, endogenous Sec61β was depleted by the transfection of siRNA. As expected, the moderate ER stress caused by Sec61β depletion and judged by *XBP1* splicing and PERK phosphorylation was alleviated when Sec61β-HA was expressed at an equivalent level (Fig. 4A). Notably, transfection of control siRNA or tetracycline treatment *per se* did not trigger ER stress (Fig. 4A). RFP-Sec61β, which does not bind to microtubules, failed to rescue Sec61β-related ER stress (Fig. 4B). However, the chimera MTB^Climp63^-TM^Sec61β^ was able to partially replace Sec61β, maintaining ER proteostasis (Fig. 4B). When we depolymerized microtubules using nocodazole (Fig. S3A), presumably abolishing ER-microtubule interactions, weak ER stress was detected (Fig. S3B and S3C).

**Figure 5:**
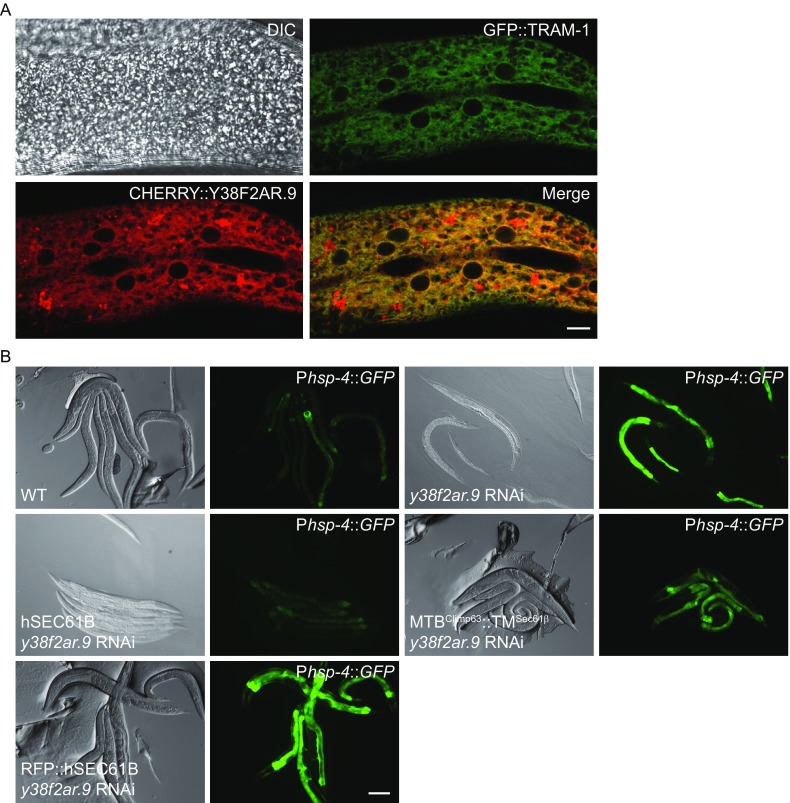
**The function of Sec61β is essential and conserved in** ***C***. ***elegans***. (A) Fluorescent micrographs of hypodermal cells in *gfp*::*tram-1* worms expressing CHERRY::Y38F2AR.9. DIC, differential interference contrast. Scale bar: 5 μm. (B) The *y38f2ar*.*9* RNAi was injected into worms carrying P*hsp-4*::*GFP*, and GFP fluorescence was assessed. For rescue assay, *y38f2ar*.*9* RNAi was injected into P*hsp-4*::*GFP* ER stress reporter worms carrying human SEC61B, MTB^Climp63^-TM^Sec61β^ or RFP-hSEC61B, and GFP fluorescence was assessed. Scale bar: 100 μm

To confirm the microtubule-binding role of Sec61β in a physiological setting, we monitored the levels of ER stress in *C*. *elegans* upon the expression of various Sec61β constructs. Elevation of the P*hsp-4*::*GFP* reporter caused by depletion of Y38F2AR.9 was efficiently inhibited by the expression of human Sec61β under a ubiquitous *nfya-1* promoter, which resists RNAi treatments (Fig. 5B). ER stress was partly suppressed when the chimera MTB^Climp63^-TM^Sec61β^ was introduced, but not when RFP-Sec61β was used (Fig. 5B). Interestingly, vectors containing human SEC61B could only be injected at very low amounts in the rescue experiments, suggesting that the Sec61β level needs to be tightly regulated. Taken together, these results confirm that the maintenance of ER homeostasis is likely linked to interactions between Sec61β and microtubules.

**Figure 6:**
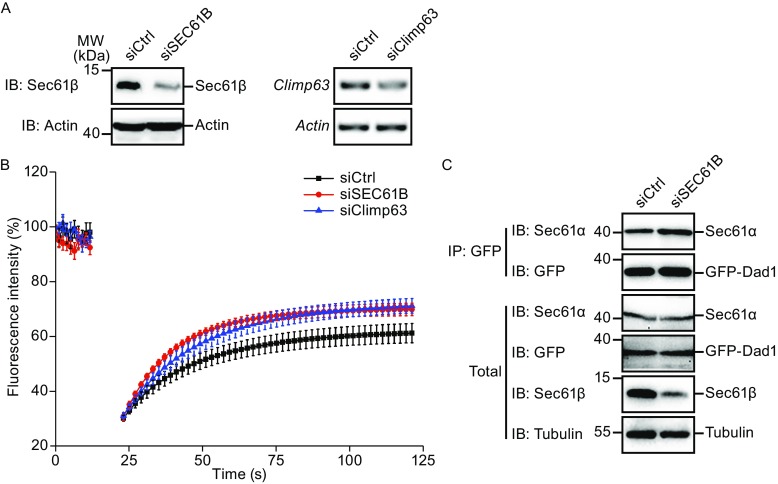
**Depletion of Sec61β improves the lateral mobility of translocon complexex**. (A) M3/18 cells transfected with siRNA of Sec61β or Climp63 for 48 h were lyzed for analysis after FRAP. The levels of Sec61β were determined by Western blotting, with actin as a loading control. *Climp63* and *Actin* by RT-PCR were resolved by agarose gel. (B) M3/18 cells in (A) were analyzed by fluorescence recovery after photobleaching assay. The initial fluorescence intensity was set at 100%. Data are mean ± SEM. (*n* =7 for control group, 6 for siSEC61B group and 5 for siClimp63 group). (C) M3/18 cells were transfected with siRNA of Sec61β for 48 h. The lysates were precipitated with anti-GFP antibody. The levels of indicated proteins were analyzed with Western blotting

ER-microtubule interaction plays a role in regulating translocon mobility (Nikonov et al., 2007). To test whether Sec61β has similar effect, we utilized M3/18 cells, in which GFP-Dad1 (a subunit of the oligosaccharyltransferase) reflects the lateral mobility of the translocon by being part of the complex (Nikonov et al., 2002). The system was first validated by FRAP analysis of GFP-Dad1 upon depletion of Climp63 (Fig. 6A); as previously reported, the mobility of translocon was increased (Fig. 6B). Consistently, depletion of Sec61β promoted moving of the translocon (Fig. 6A and 6B) without compromising Sec61α-Dad1 association (Fig. 6C). These results suggest that microtubule binding by Sec61β likely limits translocon mobility.

**Figure 7:**
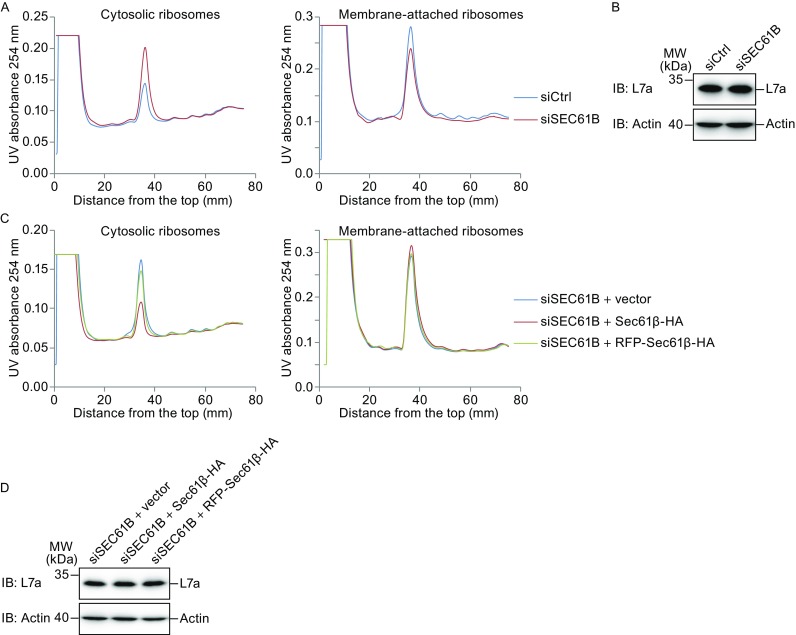
**Sec61β stabilizes membrane attachment of ribosomes**. (A) Polysome profiles of U2OS cells transfected control siRNA (siCtrl) or siSEC61B#2. (B) Total lysates from (A) were analyzed by Western blotting, with actin as a loading control. (C) As in (A), but U2OS cells co-transfected siSEC61B#2 and indicated plasmids. (D) As in (B), but from cells in (C). For all of them, the trends are similar among at least two independent repeats

Because co-translational translocation requires a stable association between the translating ribosome and translocon, we further tested whether microtubule binding by Sec61β regulates ribosome-translocon complex formation. Cytosolic ribosomes were collected after plasma membrane permeablilization, with the remaining ribosomes considered membrane-bound (i.e., ER-associated through interactions with translocons). When ribosomal profiling was analyzed using density gradients, Sec61β-depleted cells contained increased cytosolic ribosomes and decreased membrane-bound ribosomes compared to cells treated with control siRNA (Figs. 7A and S4A), even though the total amount of ribosomes judged by immunoblotting of ribosomal protein L7a remained unchanged (Fig. 7B). Consistently, RFP-tagged Sec61β failed to restore membrane attachment of ribosomes (Fig. 7C, 7D, and S4B), but the chimera MTB^Climp63^-TM^Sec61β^ succeeded (Fig. S4C–E). These results suggest that Sec61β may facilitate the attachment of ribosomes to translocons using its microtubule-binding ability.

## Discussion

Our results characterize a previously unidentified activity of the cytosolic domain of Sec61β. We show that Sec61β interacts directly with microtubules, and is involved in the maintenance of ER homeostasis. Specifically, depletion of Sec61β causes ER stress in both *C*. *elegans* and mammalian cells, and rescue of the stress appear to associate with the microtubule-binding activity, even when the cytosolic domain of Sec61β is replaced by the MTB domain of Climp63. Our results also suggest that the microtubule-binding activity of Sec61β likely stabilizes ribosome-translocon interactions. These findings provide important insights into the physiological role of the ER-microtubule association mediated by Sec61β.

Previous work on Sec61β focused mainly on its TM region. As part of the translocon, the TM of Sec61β facilitates the organization of the translocon-associated complex, including the interactions with signal peptidase and Sec62/63 (Kalies et al., 1998; Meyer et al., 2000). In yeast, the growth defects caused by double deletion of *SBH1* and *SBH2*, paralogs of Sec61β, can be restored by expression of only the TM region of yeast or human Sec61β (Feng et al., 2007; Leroux and Rokeach, 2008). Whether the translocation role associated with the TM of Sec61β is more important in other eukaryotic organisms is yet to be determined.

The cytosolic domain of Sec61β has been proposed to interact with ribosomes or exocyst complexes (Levy et al., 2001; Lipschutz et al., 2003; Toikkanen et al., 2003). Because Sec61β integrates into the ER membrane as a tail-anchored protein, it also frequently associates with cytosolic chaperones before membrane insertion (Abell et al., 2007). Our results add microtubules to the list of cytSec61β binding partners. Overexpression of Sec61β causes bundling between the ER and microtubules, endogenous Sec61β precipitates with reassembled microtubules, and purified cytSec61β co-sediments with microtubules formed *in vitro* using purified tubulin. The interaction requires the middle region of cytSec61β, which has a poorly conserved sequence but bears several basic residues and may directly engage tubulin, which contains an acidic tail. Interestingly, the interaction is inhibited when a RFP tag is added to the N-terminus of Sec61β, explaining why this activity was not seen previously (Sec61β is most commonly used in the form of a GFP/RFP fusion protein as an ER marker for live cell imaging).

The ER-microtubule association is thought to play a key role in ER morphogenesis and positioning (Goyal and Blackstone, 2013; Terasaki et al., 1986). However, due to the redundancy of microtubule-binding proteins on the ER, individual depletion rarely causes the morphological ER defects that occur when microtubules are mostly depolymerized. Consistently, knocking down Sec61β does not alter ER morphology, but causes ER stress. Restoration of the ER homeostasis is only achieved when wild type Sec61β or the chimera MTB^Climp63^-TM^Sec61β^ is reintroduced, implicating an involvement of ER-microtubule interaction in ER homeostasis, instead of ER shaping. Poor expression of the microtubule-binding mutants of Sec61β limits our analysis and leaves the possibility that cytSec61β is critical for other functions.

We also found that Sec61β stabilizes the ribosome-translocon association. These findings are consistent with previous reports showing that ER stress is triggered when ribosomal membrane-targeting is reduced (Gamerdinger et al., 2015). The stress is possibly caused by a shortage of necessary factors for maintaining ER homeostasis and/or mistakes resulting from premature separation of the ribosome-translocon complex. It is also likely that loss of Sec61β interferes with recently reported functional linkage between translocon and IRE1 (Plumb et al., 2015; Sundaram et al., 2017), which in turn triggers UPR. However, IRE1 engages translocon mainly through its TM segment, whether cytSec61β is involved remains to be investigated.

Another ER sheet-enriched protein that interacts with microtubules is Climp63. The microtubule association by Climp63 has been proposed to regulate the mobility of the translocon and, thus, may indirectly regulate the stability of the translocating complex. We found that depletion of either protein increases translocon mobility. However, depletion of Climp63 does not cause ER stress as seen with Sec61β, suggesting that the microtubule-binding ability of Sec61β has a direct impact on translocon, and that of Climp63 may have other functions, such as positioning ER sheets in the perinuclear region.

Our findings partly explain why Sec61β is essential in higher eukaryotes. Sec61β has also been reported to have specific roles, such as regulating the transport of Gurken, an EGF homolog in *Drosophila*, to the plasma membrane (Kelkar and Dobberstein, 2009), and its involvement in the inner nuclear membrane transport of EGFR (Liao and Carpenter, 2007; Liao and Carpenter, 2009; Wang et al., 2010). Whether these activities are associated with the microtubule-binding ability identified here remains to be tested. Notably, *C*. *elegans* lines expressing Sec61β can only be obtained when vectors are injected at a very low level, implying that overexpression of Sec61β is hazardous and its level needs to be fine-tuned in higher organisms.

## Materials and methods

### Constructs

Fragments of human SEC61B were amplified from its cDNAs and connected using overlap PCR to generate truncations and chimeras. MTB domain of Climp63 (residues 1–80) was amplified from a plasmid coding mouse Climp63. For mammalian cell expression, the indicated fragments were PCR-amplified with an N- or C-terminal HA tag and ligated into pcDNA4/TO or pcDNA5/TO vector. The plasmid mEmerald-Ensconsin is a gift from Dong Li’s lab. For *Caenorhabditis elegans* expression, *y38f2ar.9* containing its 3′-untranslated region (3′-UTR) was amplified and subcloned into pPD49.26 vector with the promoter of *hyp7* and *cherry* on the N terminus. The rescue fragments, which were the same with those in mammalian cells, were subcloned into pPD49.26 vector with the promoter of *nfya-1*. For protein purification, the indicated fragments were subcloned into pSUMO vector.

### siRNAs, shRNAs and gRNA for indicated proteins

SiRNA oligonucleotides targeting Sec61β (Wang et al., 2010), REEP1 and nonspecific siRNA control were purchased from RiboBio. The siRNA sequences are as following: Sec61β siRNA #1, 5′-GCAAGUACACUCGUUCGUA-3′; Sec61β siRNA #2, 5′-CUGUAAGCUUGCUGUUUUA-3′; Sec61β siRNA for M3/18 cells, 5′-GCAAGUACACACGCUCAUA-3′; REEP1 siRNA #1, 5′-GGCUGGUGGUGCUUAUAUU-3′; REEP1 siRNA #2, 5′-CCUCCUUUACAGGAAGUUU-3′; Climp63 siRNA for M3/18 cells, 5′-CCAAGUCCAUCAAUGACAA-3′ (Nikonov et al., 2007). The shRNA coding plasmids were purchased from Sigma. The shRNA coding sequences are as following: Sec61β shRNA #1, 5′-CCGGCAGTATTGGTTATGAGTCTTCCTCGAGGAAGACTCATAACCAATACTGTTTTTTG-3′; Sec61β shRNA #2, 5′-CCGGCCCAACATTTCTTGGACCAAACTCGAGTTTGGTCCAAGAAATGTTGGGTTTTTTG-3′. The sequence of gRNA used in U2OS Climp63 deletion cell line is 5′-CGCCGCGCCCGCCATGCCCT-3′.

### Cell culture, transfection, and co-immunoprecipitation

COS-7 cells (ATCC), U2OS cells (ATCC), MEF cells and M3/18 cells (a gift from Gert Kreibich’s group) were maintained in Dulbecco’s Modified Eagle’s medium (DMEM; Invitrogen) supplemented with 10% fetal bovine serum (Gibico) at 37 °C (but M3/18 cells at 39.5 °C) in 5% CO_2_. Flp-In^TM^ T-REx^TM^-293 (Invitrogen) expression cell lines were generated following Invitrogen’s protocol and maintained in DMEM (Invitrogen) with 10% fetal bovine serum (HyClone), 2 mmol/L L-glutamine (Invitrogen), 15 μg/mL Blasticidin, 100 μg/mL Hygromycin and Penicillin-Streptomycin (Invitrogen) at 37 °C in 5% CO_2_. The Sec61β shRNA stable cell lines were generated following the pLKO.1 protocol (Addgene). Transfections were performed using TurboFect (Thermo) for plasmids and Lipofectamine RNAiMAX (Invitrogen) for siRNAs according to the manufacturer’s instructions. For co-immunoprecipitation experiments, 45% confluent M3/18 cells were transfected with indicated siRNAs and harvested 48 h later in IP buffer (25 mmol/L HEPES pH 7.4, 150 mmol/L KAC, 2 mmol/L Mg(AC)_2_ and protease inhibitors) containing 1% digitonin. Cell lysates were incubated with anti-GFP agarose (MBL) for 2 h at 4 °C. Washed precipitates were separated by SDS-PAGE and immunoblotted with anti-GFP and anti-Sec61β antibodies (Sigma).

### Strains

*Caenorhabditis elegans* were cultured according to standard techniques (Brenner, 1974). The following strains were used in this work: *zcIs4* (P*hsp-4*::*GFP*) and *qxIs439* (P*hpy7*::*gfp*::*tram-1*). All experiments were performed at 20 °C unless otherwise noted.

### Immunofluorescence and confocal microscopy

COS-7 cells or U2OS cells were fixed with 4% paraformaldehyde (PFA) in PBS for 25 min, permeabilized with 0.1% Triton X-100/PBS for 10 min, and blocked with 3% BSA for 1 h at room temperature. Fixed cells were then incubated with primary antibodies for 1 h at room temperature or overnight at 4 °C, including rabbit anti-calreticulin (Abcam; 1:800), mouse anti-Tubulin (Thermo; 1:200), rabbit anti-Tubulin (Abcam; 1:1000), mouse anti-HA (Sigma; 1:500), rabbit anti-HA (Abcam; 1:1000), mouse anti-GM130 (BD; 1:500) and mouse anti-TOM20 (BD; 1:500), followed by incubation with various fluorophore-conjugated secondary antibodies (Alexa Fluor 488-conjugated anti-rabbit or mouse, Alexa Fluor 568-conjugated anti-mouse or rabbit, Invitrogen) for 1 h at room temperature. All images were captured on Leica TCS SP5 or Zeiss LSM700 confocal microscope with a 63× objective. Brightness and contrast were adjusted across the entire image using Adobe Photoshop.

### Microtubule co-sedimentation assay

For *in vivo* assay, following 10 min’s incubation on ice in 500 μL MME buffer (100 mmol/L MES pH 6.8, 1 mmol/L MgCl_2_, 1 mmol/L EGTA, 1 mmol/L DTT and protease inhibitor) containing 1% Trition X-100, COS-7 cells from a 6-cm dish were homogenized by the tight-fitted Dounce homogenizer for 150 strokes, and then placed on ice for 30 min before centrifugation for 10 min at 20,000 × *g*. The supernatant was added to 30 μmol/L paclitaxel (Sigma) and 1mmol/L GTP (Sigma) in a 100 μL reaction volume followed by incubation at 37 °C for 30 min, and the mixture was loaded over the MME Cushion buffer (100 mmol/L MES pH 6.8, 1 mmol/L MgCl_2_, 1 mmol/L EGTA, 1 mmol/L DTT, 20% glycerol and protease inhibitors) containing 30 μmol/L paclitaxel and centrifuged at 32 °C for 50 min at 100,000 × *g*. The negative control was incubated on ice without GTP and paclitaxel, and centrifuged at 4 °C for 50 min at 100,000 × *g*. The pellet (containing microtubules and associated proteins) and supernatant fractions were then collected and examined by Western blotting. For *in vitro* assay, microtubules were assembled with 5 μmol/L bovine brain tubulin (Cat. #TL238, Cytoskeleton; a gift from Jun Zhou’s lab) in 100 μL BRB80 buffer (80 mmol/L PIPES pH 6.8, 1 mmol/L EGTA, 1 mmol/L MgCl_2_, 5% glycerol and protease inhibitor) in the presence of 1 mmol/L GTP at 37 °C for 30 min, and then added to 30 μmol/L paclitaxel and 1.5 μmol/L purified protein (centrifuged at 4 °C for 10 min at 100,000 × *g*) followed by incubation for another 20 min at 37 °C. The reaction mixture was then analyzed as above.

### Protein expression, purification, and pull-down assay

The cytosolic domain of human Sec61β and its Δ20–44 truncation fused with an N-terminal cleavable His-SUMO tag followed by an HA tag were expressed in *Escherichia coli*. The cells were lysed in lysis buffer (50 mmol/L Tris pH 8.0, 300 mmol/L NaCl, 2 mmol/L β-mercaptoethanol and 20 mmol/L imidazole) containing 1 mmol/L PMSF. The proteins were isolated with Ni-NTA, washed, and eluted with 300 mmol/L imidazole in lysis buffer. The His-SUMO tag was cleaved with His-tagged SUMO protease Ulp1p and removed by Ni-NTA chromatography followed by gel filtration. For pull-down assay, 5 μmol/L tubulin and 1.5 μmol/L purified protein were precipitated with anti-HA agarose (Sigma) in IP buffer (50 mmol/L Tris pH 7.5, 150 mmol/L NaCl, 1 mmol/L EDTA, 30% glycerol and protease inhibitors) containing 1% NP-40 for 2 h at 4 °C. Washed precipitates were separated by SDS-PAGE and immunoblotted with anti-HA and anti-Tubulin antibodies (Abcam).

### RNA isolation and RT-PCR

Cells were lysed using Trizol (Invitrogen) and total RNA was collected. cDNA reverse-transcribed from poly-A mRNA was used as template for PCR with the following primers: specific for human and monkey *XBP1*, 5′-CCTTGTAGTTGAGAACCAGG-3′ and 5′-GGGGCTTGGTATATATGTGG-3′ (Szczesna-Skorupa et al., 2004); specific for mouse *XBP1*, 5′-CCTTGTGGTTGAGAACCAGG-3′ and 5′-GAGGCTTGGTGTATACATGG-3′; specific for monkey *REEP1* #1, 5′-TTGTAGCCTGGCTGCTGTCTCC-3′ and 5′-AAGCAGCCATCACAGCCGCTG-3′; monkey *REEP1* #2, 5′-GGACAGGGTGCCTTATCAG-3′ and 5′-ACTCCTGGACATCTTAGGCTG-3′; for monkey *GAPDH*, 5′-GAAGGTGAAGGTCGGAGTCA-3′ and 5′-GAAGATGGTGATGGGATTTC-3′. PCR products were resolved on a 2.5% agarose/1× TAE gel. A hybrid amplicon species consisting of unspliced *XBP1* annealed to spliced *XBP1* was also produced through the PCR and was visible as a slower migrating band above the unspliced amplicon (Li et al., 2010).

### RNAi and rescue assay in *C*. *elegans*

To determine the localization of Y38F2AR.9, the *cherry*::*y38f2ar*.*9* construct was injected into *gfp*::*tram-1* animals at the concentration of 1ng/μL and pRF4(*rol-6*[*su1006*]) was co-injected. The F1 animals were checked with Zeiss LSM710 META confocal microscope. For RNAi injection experiments, single-stranded RNA was transcribed from T7- and SP6-flanked PCR templates. ssRNAs were then annealed and injected into animals carrying P*hsp-4*::*GFP*. The F1 animals were checked. The DNA template used for RNA synthesis was *y38f2ar*.*9* (YAC Y38F2AR: nt 56335–56636). For rescue assay, the indicated construct was injected into the *Phsp*-*4*::*GFP* worms together with pRF4(*rol-6*[*su1006*]) and *y38f2ar*.*9*RNAi was then injected into the F2 animals. The F3 animals were checked with Zeiss LSM710 META confocal microscope.

### Fluorescence recovery after photobleaching

M3/18 cells were seeded and grown overnight at 39.5 °C on glass-bottom 35-mm tissue culture dishes (MatTek) in complete growth medium. After siRNA transfection for 48 h, the cells were ready for FRAP at 39.5 °C in 5% CO_2_. FRAP experiments were performed on a Leica TCS SP5 confocal microscope as previously described (Nikonov et al., 2002).

### Isolation of cytosolic and membrane-bound ribosomes

U2OS cells were seeded on two 15-cm plates for each group and allowed to grow to 100% confluence. 100 μg/mL of cycloheximide was added to the cells for 15 min before harvest as described (Zhang and Zhou, 2012). Cells were resuspended in Polysome Extraction Buffer (PEB; 20 mmol/L Tris pH 7.5, 50 mmol/L KCl, 10 mmol/L MgCl_2_, 1 mmol/L DTT, 100 μg/mL CHX, 500 U/mL RNasin and protease inhibitors) containing 0.008% (*w*/*v*) digitonin, incubated for 5 min on ice (Gamerdinger et al., 2015) and centrifuged at 800 rpm for 4 min. The supernatant containing cytosolic ribosomes was collected. After two washing steps in PEB buffer, membrane-bound ribosomes were released by incubating pellets in PEB buffer supplemented with 1% (*v*/*v*) Triton X-100 for 30 min on ice. After centrifugation at 14,000 rpm for 30 min, the supernatant containing membrane-bound ribosomes was collected. The cytosolic and membrane fractions were then loaded on a 10%–50% linear sucrose gradient and sedimented in a SW41 rotor at 247,600 × *g* for 2 h at 4 °C. The gradients were fractionated using a piston gradient fractionator (BioComp Instruments, Fredericton, NB, Canada) and UV absorbance at 254 nm was monitored by a UV-Monitor (BioRad, Hercules, CA).

## Electronic supplementary material

Below is the link to the electronic supplementary material.

Supplementary material 1 (DOCX 16966 kb)

Supplementary material 2 (DOCX 7571 kb)

Supplementary material 3 (DOCX 10867 kb)

Supplementary material 4 (DOCX 1120 kb)

Supplementary material 5 (DOCX 107 kb)
