# Supplementary material for: Sec61β facilitates the maintenance of endoplasmic reticulum homeostasis by associating microtubules
Source: Protein Cell. 2017 Nov 22;9(7):616–28. doi: 10.1007/s13238-017-0492-5 (PMC6019657; doi:10.1007/s13238-017-0492-5)
Supplement: Supplementary file 1 — Supplementary material 1 (DOCX 16966 kb) [file 13238_2017_492_MOESM1_ESM.docx]

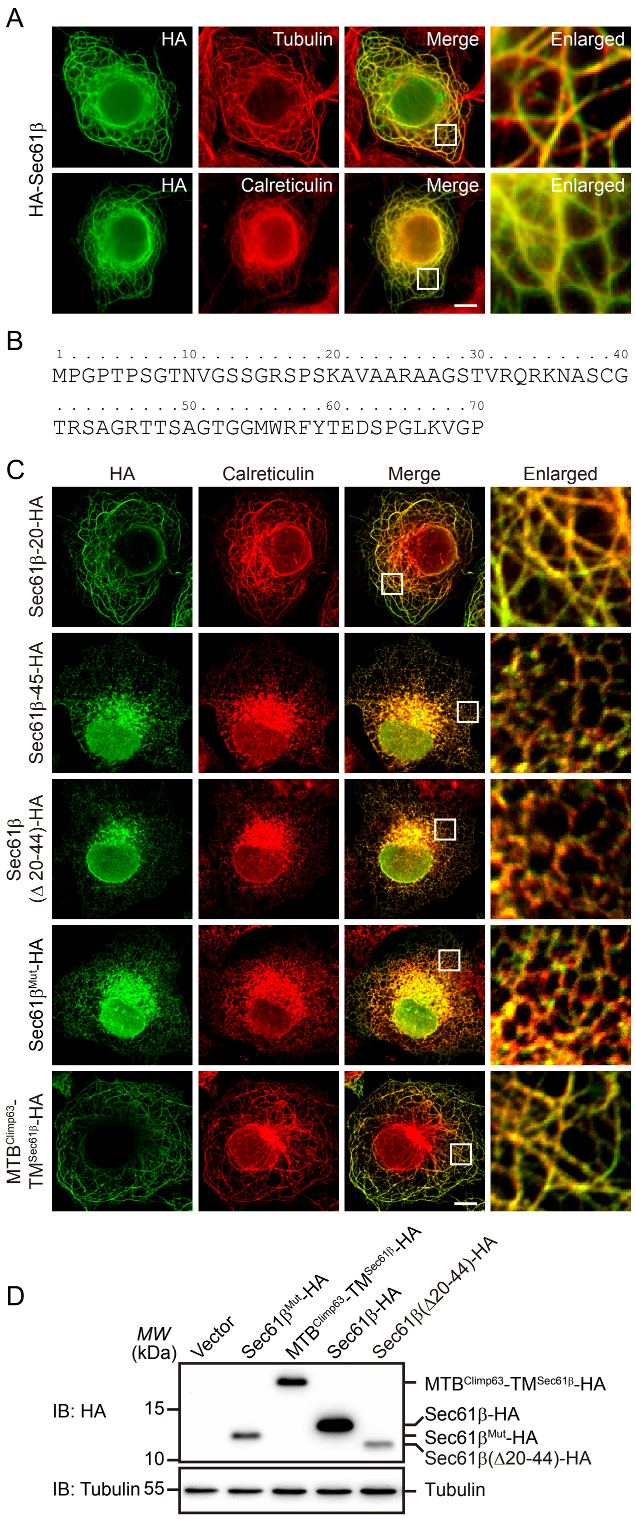

Figure S1. The middle region of cytSec61β is required for microtubule binding.
(A) COS-7 cells transfected with HA-Sec61β were immunostained for HA-epitope (green) and endogenous Tubulin or luminal ER protein, Calreticulin (red), and visualized by fluorescent confocal microscopy. Insets show the enlargement of the indicated area. Scale bar: 10 μm.
(B) The amino acid sequence of cytSec61β.
(C) The localization of proteins shown in Figure 1E were visualized with anti-HA (green) and anti-Calreticulin (red) antibodies by indirect immunofluorecence and confocal microscopy. Insets show the enlargement of the indicated area. Scale bar: 10 μm.
(D) Expression of HA-tag proteins used in Figure 1E and S1C was analyzed by Western blotting, with tubulin as a loading control.
