# Supplementary material for: Sec61β facilitates the maintenance of endoplasmic reticulum homeostasis by associating microtubules
Source: Protein Cell. 2017 Nov 22;9(7):616–28. doi: 10.1007/s13238-017-0492-5 (PMC6019657; doi:10.1007/s13238-017-0492-5)
Supplement: Supplementary file 2 — Supplementary material 2 (DOCX 7571 kb) [file 13238_2017_492_MOESM2_ESM.docx]

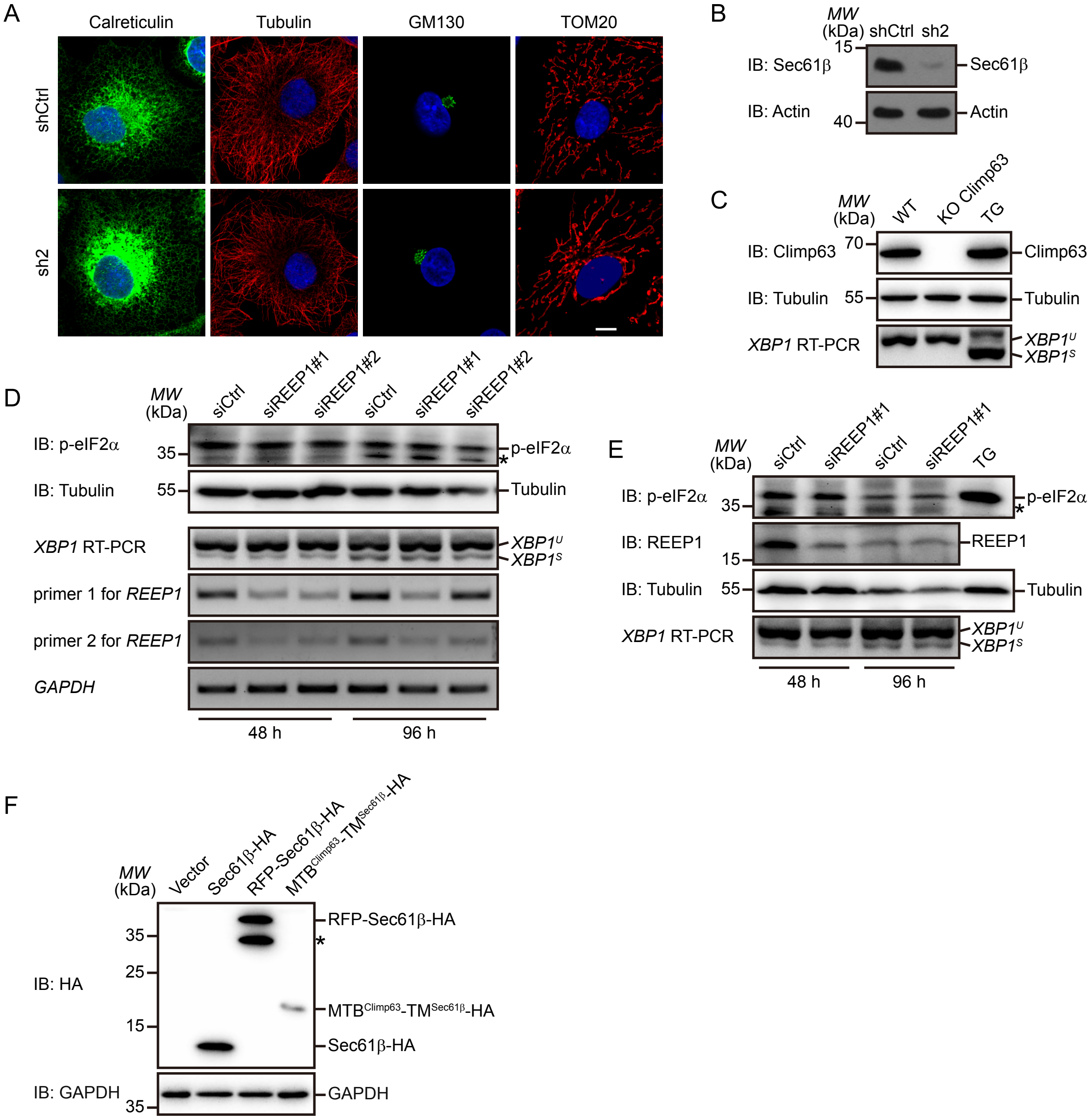

Figure S2. Depletion of Sec61β causes ER stress.

(A) COS-7 cells infected with shRNA-expressing viruses were immunostained for Calreticulin, Tubulin, GM130 and TOM20 respectively (with DAPI staining), and visualized by confocal microscopy. Scale bar: 10 μm.

(B) COS-7 cells were infected with shRNA-expressing viruses, and the level of Sec61β determined by Western blotting.

(C) The levels of Climp63 in indicated cells was determined by Western blot, and the unspliced (U) *XBP1* and spliced (S) *XBP1* by RT-PCR of *XBP1* mRNA were resolved by agarose gel.

(D) COS-7 cells were transfected with siRNAs of REEP1 for 48 hours or 96 hours. The levels of phosphorylated eIF2α were determined by Western blotting, and the *REEP1*, *GAPDH*, unspliced *XBP1* and spliced *XBP1* by RT-PCR were resolved by agarose gel. Asterisk (*) indicates a nonspecific band.

(E) MEF cells were transfected with siRNAs of REEP1 for 48 hours or 96 hours. The levels of phosphorylated eIF2α and REEP1 were determined by Western blotting, and the unspliced *XBP1* and spliced *XBP1* by RT-PCR of *XBP1* mRNA were resolved by agarose gel. TG treated group was the positive control. Asterisk (*) indicates a nonspecific band.

(F) Lysates of generated Flp-In-293 cell lines were analyzed with Western blotting. The band with asterisk (*) may be degraded RFP-Sec61β-HA.
