# Supplementary material for: Sec61β facilitates the maintenance of endoplasmic reticulum homeostasis by associating microtubules
Source: Protein Cell. 2017 Nov 22;9(7):616–28. doi: 10.1007/s13238-017-0492-5 (PMC6019657; doi:10.1007/s13238-017-0492-5)
Supplement: Supplementary file 4 — Supplementary material 4 (DOCX 1120 kb) [file 13238_2017_492_MOESM4_ESM.docx]

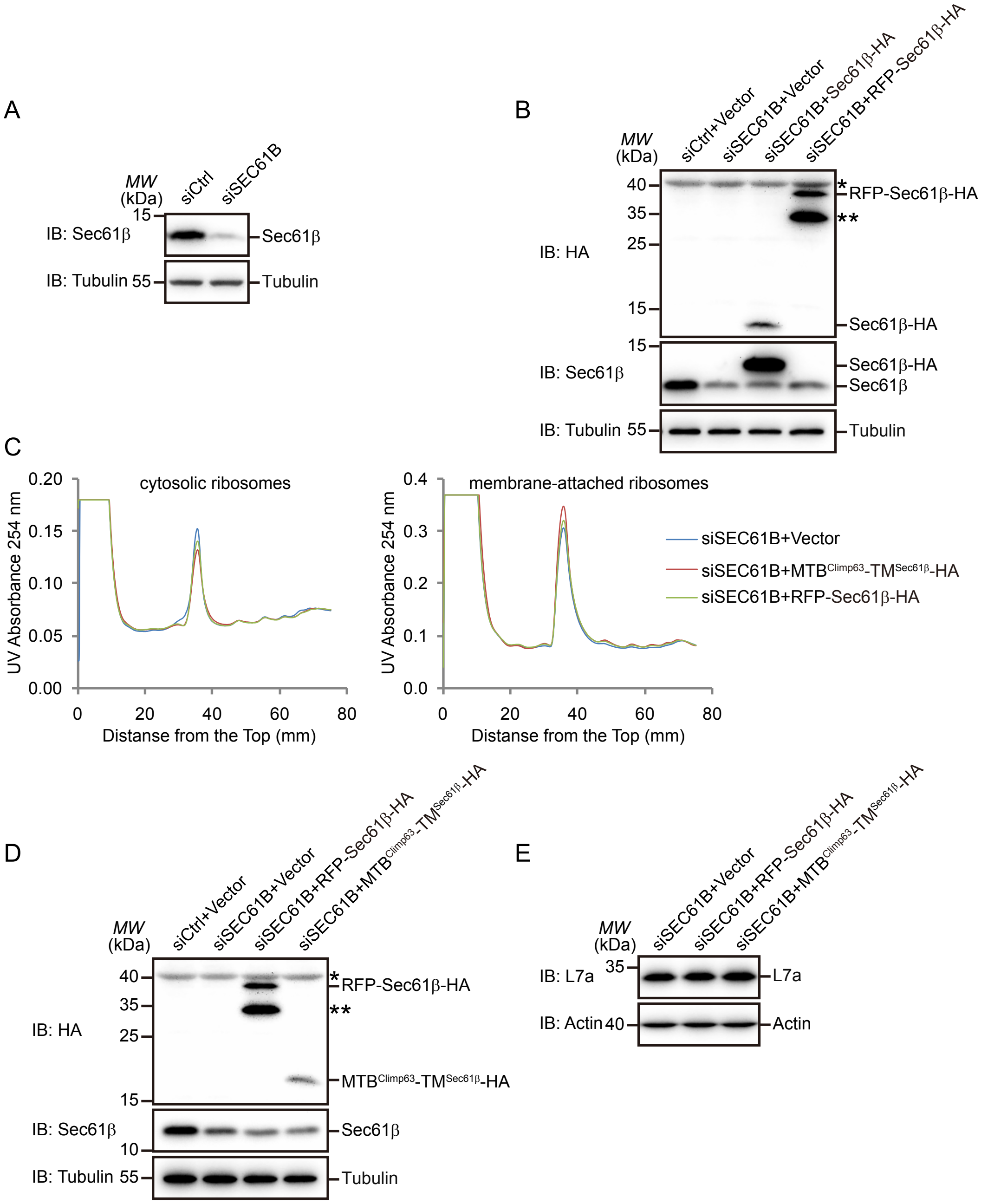

Figure S4. Controls for polysome profiles.

(A) Expression of indicated proteins used in Figure 7A was analyzed by Western blotting, with tubulin as a loading control.

(B) As in (A), but proteins used in Figure 7C. As the SW41 rotor can only accommodate 6 samples, so the control group was not done for polysome profiles. Asterisk (*) indicates a nonspecific band. The band with asterisk (**) may be degraded RFP-Sec61β-HA.

(C) Polysome profiles of U2OS cells co-transfected siSEC61B#2 and indicated plasmids.

(D) As in (B), but proteins used in (C).

(E) Total lysates from (C) were analyzed by Western blotting, with actin as a loading control.
