# Supplementary material for: Sec61β facilitates the maintenance of endoplasmic reticulum homeostasis by associating microtubules
Source: Protein Cell. 2017 Nov 22;9(7):616–28. doi: 10.1007/s13238-017-0492-5 (PMC6019657; doi:10.1007/s13238-017-0492-5)
Supplement: Supplementary file 5 — Supplementary material 5 (DOCX 107 kb) [file 13238_2017_492_MOESM5_ESM.docx]

**Supplemental Information**

**Figure Legends**

**Figure S1**. The middle region of cytSec61β is required for microtubule binding.

(A) COS-7 cells transfected with HA-Sec61β were immunostained for HA-epitope (green) and endogenous Tubulin or luminal ER protein, Calreticulin (red), and visualized by fluorescent confocal microscopy. Insets show the enlargement of the indicated area. Scale bar: 10 mm. (B) The amino acid sequence of cytSec61β. (C) The localization of proteins shown in Figure 1E were visualized with anti-HA (green) and anti-Calreticulin (red) antibodies by indirect immunofluorecence and confocal microscopy. Insets show the enlargement of the indicated area. Scale bar: 10 μm. (D) Expression of HA-tag proteins used in Figure 1E and S1C was analyzed by Western blotting, with tubulin as a loading control.

**Figure S2**. Depletion of Sec61β causes ER stress.

(A) COS-7 cells infected with shRNA-expressing viruses were immunostained for Calreticulin, Tubulin, GM130 and TOM20 respectively (with DAPI staining), and visualized by confocal microscopy. Scale bar: 10 μm. (B) COS-7 cells were infected with shRNA-expressing viruses, and the level of Sec61β determined by Western blotting. (C) The levels of Climp63 in indicated cells was determined by Western blot, and the unspliced (U) XBP1 and spliced (S) XBP1 by RT-PCR of XBP1 mRNA were resolved by agarose gel. (D) COS-7 cells were transfected with siRNAs of REEP1 for 48 hours or 96 hours. The levels of phosphorylated eIF2α were determined by Western blotting, and the REEP1, GAPDH, unspliced XBP1 and spliced XBP1 by RT-PCR were resolved by agarose gel. Asterisk (*) indicates a nonspecific band. (E) MEF cells were transfected with siRNAs of REEP1 for 48 hours or 96 hours. The levels of phosphorylated eIF2α and REEP1 were determined by

Western blotting, and the unspliced XBP1 and spliced XBP1 by RT-PCR of XBP1 mRNA were resolved by agarose gel. TG treated group was the positive control. Asterisk (*) indicates a nonspecific band. (F) Lysates of generated Flp-In-293 cell lines were analyzed with Western blotting. The band with asterisk (*) may be degraded RFP-Sec61b-HA.

**Figure S3**. Depolymerization of microtubule induces mild ER stress.

(A) U2OS cells were treated with different concentrations (μg/mL) of nocodazole for 30 minutes to depolymerize microtubules. The morphology of the microtubule and ER were determined by immunostaining with tubulin (green) and calreticulin (red), respectively, and visualized by fluorescence confocal microscopy. Scale bar: 10 μm.

(B) Unspliced (U) XBP1 and spliced (S) XBP1 using samples in (A) were resolved by agarose gel. (C) As in (B), but for 2 hours.

**Figure S4**. Controls for polysome profiles.

(A) Expression of indicated proteins used in Figure 7A was analyzed by Western blotting, with tubulin as a loading control. (B) As in (A), but proteins used in Figure 7C. As the SW41 rotor can only accommodate 6 samples, so the control group was not done for polysome profiles. Asterisk (*) indicates a nonspecific band. The band with asterisk (**) may be degraded RFP-Sec61b-HA. (C) Polysome profiles of U2OS cells co-transfected siSEC61B#2 and indicated plasmids. (D) As in (B), but proteins used in (C). (E) Total lysates from (C) were analyzed by Western blotting, with actin as a loading control.
