# Supplementary material for: Sec61β facilitates the maintenance of endoplasmic reticulum homeostasis by associating microtubules
Source: Protein Cell. 2017 Nov 22;9(7):616–28. doi: 10.1007/s13238-017-0492-5 (PMC6019657; doi:10.1007/s13238-017-0492-5)
Supplement: Supplementary file 3 — Supplementary material 3 (DOCX 10867 kb) [file 13238_2017_492_MOESM3_ESM.docx]

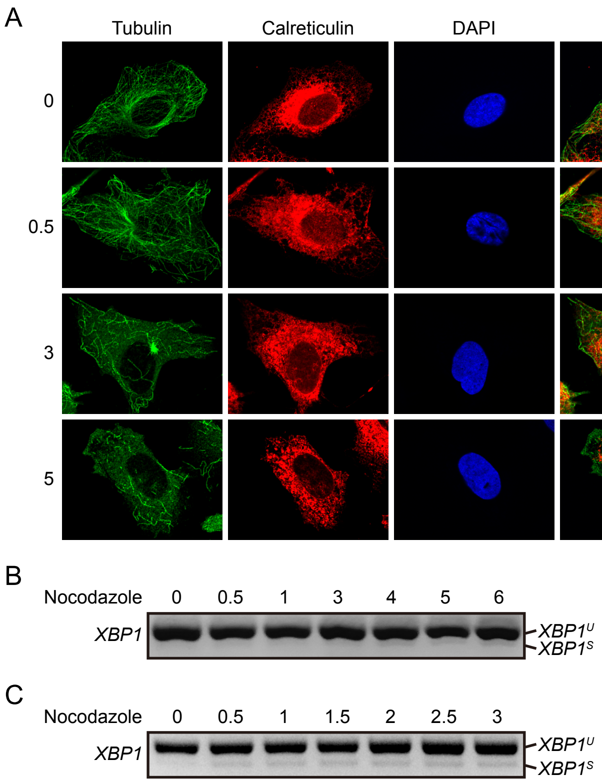

Figure S3. Depolymerization of microtubule induces mild ER stress.
(A) U2OS cells were treated with different concentrations (μg/mL) of nocodazole for 30 minutes to depolymerize microtubules. The morphology of the microtubule and ER were determined by immunostaining with tubulin (green) and calreticulin (red), respectively, and visualized by fluorescence confocal microscopy. Scale bar: 10 μm.
(B) Unspliced (U) *XBP1* and spliced (S) *XBP1* using samples in (A) were resolved by agarose gel.
(C) As in (B), but for 2 hours.
